# Study protocol of a randomized controlled trial evaluating the Prime Time Sister Circles (PTSC) program's impact on hypertension among midlife African American women

**DOI:** 10.1186/s12889-021-10459-8

**Published:** 2021-03-29

**Authors:** Chidinma A. Ibe, Danielle R. Haywood, Ciana Creighton, Yidan Cao, Angel Gabriel, Hossein Zare, Wehmah Jones, Manshu Yang, Michele Balamani, Marilyn Gaston, Gayle Porter, Denise L. Woods, Darrell J. Gaskin

**Affiliations:** 1 Division of General Internal Medicine, Department of Medicine, Johns Hopkins University School of Medicine, Baltimore, MD USA; 2 Department of Health, Behavior and Society, Johns Hopkins Bloomberg School of Public Health, Baltimore, MD USA; 3 Johns Hopkins Center for Health Disparities Solutions, Johns Hopkins Bloomberg School of Public Health, Baltimore, MD USA; 4 Trachtenberg School of Public Policy and Administration, George Washington University, Washington, DC USA; 5 Mayor’s Office of Policy, Executive Office of the Mayor, Washington, DC USA; 6 Howard University College of Medicine, Washington, DC USA; 7 Department of Health Policy and Management, Johns Hopkins Bloomberg School of Public Health, 624 North Broadway Ste 441, Baltimore, MD 21205 USA; 8 Department of Global Health Services and Administration, University of Maryland Global Campus, Adelphi, MD USA; 9 American Institutes for Research, Washington, DC USA; 10 Department of Psychology, University of Rhode Island, Kingston, RI USA; 11 The Gaston & Porter Health Improvement Center, Inc., Washington, DC USA; 12 Baraka and Associates, Largo, MD USA

**Keywords:** Peer support, Stress management, Hypertension, African American women

## Abstract

**Background:**

The Prime-Time Sister Circles® (PTSC) program is a multifaceted, community-based peer support intervention targeting African American women who are 40 to 75 years of age. It aims to reduce hypertension disparities observed among African American women by promoting adherence to antihypertensive therapies, including lifestyle modification and therapeutic regimens.

**Methods:**

The PTSC randomized controlled trial will evaluate the effectiveness of the PTSC Program on improved blood pressure control, healthcare utilization attributed to cardiovascular events, and healthcare costs. The study began in 2016 and will end in 2022. African American women who are 40–75 years old, have been diagnosed with hypertension, reside in Washington, D.C. or Baltimore, Maryland, and receive their care from Unity Health Care, a federally qualified health center in Washington, D.C., or Baltimore Medical System, a federally qualified health center in Baltimore, Maryland, are eligible to participate. Those randomized to the intervention group participate in the PTSC Program, which spans 13 weeks and comprises facilitator-led discussions, didactic training about hypertension management, and peer-based problem-solving concerning CVD risk factors and their amelioration. Blood pressure, weight, body mass index, waist circumference, self-reported adherence, physical activity, dietary practices, stress, and healthcare utilization data are collected at baseline, 13 weeks (end of the intervention), 9 months (months post-intervention), and 15 months (one year after the intervention). Healthcare costs will be computed at the end of the study. The study’s design is reported in the present manuscript, wherein we employed the SPIRIT checklist to guide its construction.

**Discussion:**

Disparities in hypertension prevalence and management observed among mid-life African American women exist as a result of a confluence of structural determinants of health. Consequently, there is a need to develop, implement, and evaluate culturally appropriate and relevant interventions that are tailored to their lived experiences. The PTSC Trial aims to assess the impact of the program on participants’ cardiovascular, psychosocial, and cost outcomes. Its results have implications for advancing the science of designing and implementing culturally relevant interventions for African American women.

**Trial registration:**

Unique identifier: NCT04371614. Retrospectively registered on April 30, 2020.

## Background

Hypertension affects approximately one-third of adults in the United States and significantly contributes to coronary heart disease, stroke, peripheral vascular disease, congestive heart failure, myocardial infarction, and renal failure [1, 2]. It is the single largest risk factor for cardiovascular mortality [3]. Non-Hispanic Black women are disproportionately encumbered by high blood pressure [4]. Recently, Chen and colleagues observed that Black women have the greatest cumulative risk of developing hypertension (86%) relative to White men (84%), Black men (85%), and White women (69%) [5]. Indeed, the prevalence of hypertension among African American (AA) women is 46.1%, the highest in the United States [6].

Hypertension disparities observed among AA women, particularly those aged 40 to 75 years of age, are rooted in a constellation of intrapersonal, interpersonal, health care system, and societal factors, whose intersection may adversely affect the degree to which adequate achievement and maintenance of blood pressure control occurs [7, 8]. Their presence suggests a strong need to identify effective strategies that attend to the confluence of unique sociocultural needs facing AA women, which may, in turn, enhance their capacity to manage their blood pressure successfully. One strategy for tackling uncontrolled hypertension among middle-aged AA women is the development and implementation of multi-component community-based interventions that promote therapeutic lifestyle changes [9, 10]. These interventions are typified by an emphasis on advancing culturally-rooted educational, behavioral, and psychosocial approaches to target modifiable CVD risk factors [11–14].

One such intervention is the Prime Time Sister Circles® (PTSC) Program, which addresses specific risk factors and barriers experienced by mid- to late-life AA women who have, or are at risk of developing, hypertension, with the goal of equipping program participants to manage their blood pressure through primary and secondary prevention strategies. There is indication to suggest that participation in the PTSC program is associated with increased physical activity; improved dietary patterns; reduced blood pressure, weight, and self-reported stress; and increased blood pressure self-monitoring, health screenings, and engagement in strategies geared toward psychosocial health [15, 16]. While previous investigations of the program employed quasi-experimental designs to discern its impact on clinical, behavioral, and psychosocial factors CVD risk factors, the use of more rigorous study designs to determine the effectiveness of the PTSC program is warranted.

Consequently, we have designed and are currently implementing a randomized controlled trial (RCT) to evaluate the association between exposure to the PTSC program and participants’ cardiovascular outcomes. The purpose of this paper is to describe this trial, the PTSC-RCT, which will ascertain the extent to which the PTSC program improves participants’ outcomes related to CVD risk factors, including education (knowledge about hypertension sequelae), behavioral (self-reported adherence), psychosocial (i.e., stress management and self-efficacy to manage blood pressure), clinical (blood pressure control, waist circumference, and BMI), and avoidable healthcare utilization (hospitalizations and inpatient admissions) attributable to CVD risk factors among AA women aged 40 to 75 years old residing in metropolitan Washington, D.C. or Baltimore, Maryland.

## Methods

**Fig. 1:**
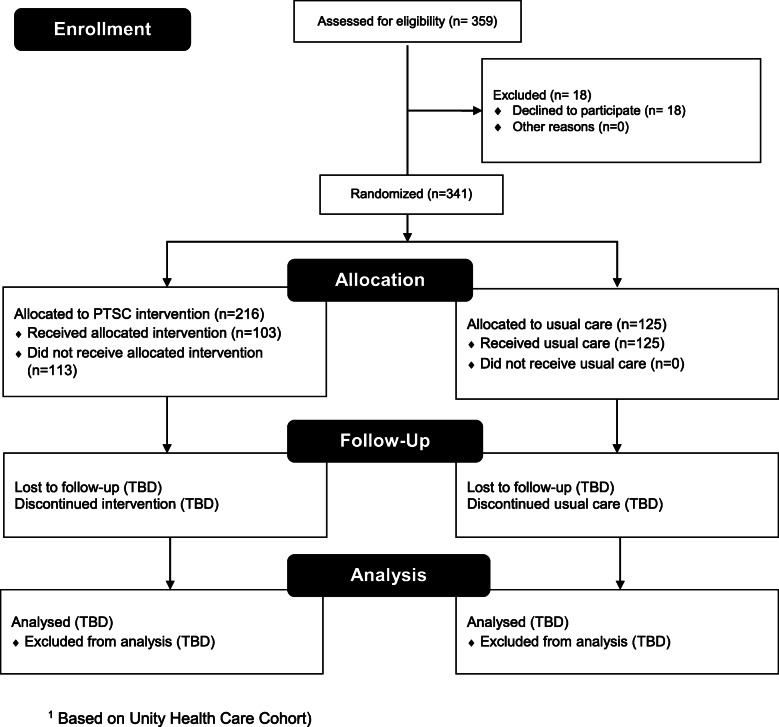
CONSORT Flow Diagram [1]

**Fig. 2:**
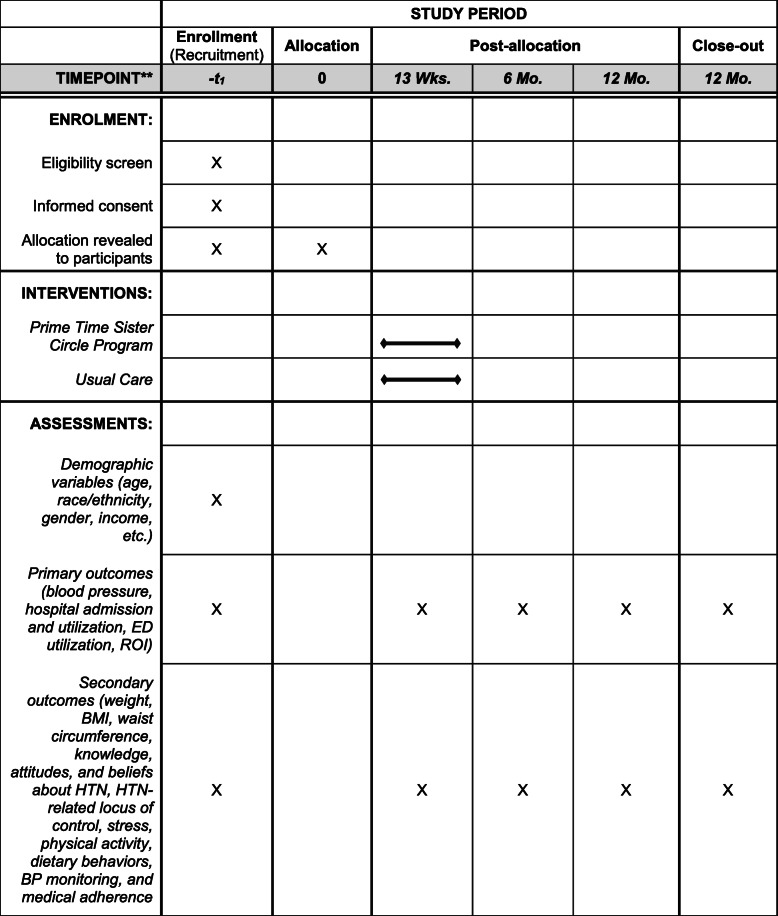
SPIRIT Flow Diagram of the PTSC-RCT Protocol

The protocol’s flow is delineated in the CONSORT Diagram (Fig. 1) and the SPIRIT Flow Diagram (Fig. 2). We provide additional details about the PTSC trial below.

### PTSC program overview

The PTSC Program is a multifaceted, theoretically-driven, community-based peer support intervention targeting AA women who are 40 to 75 years of age. It was developed by two mid-life AA women, Drs. Marilyn Gaston, a physician, and Gayle Porter, a clinical psychologist, and established through the auspices of The Gaston and Porter Health Improvement Center, Inc. (GPHIC). The GPHIC’s National Training Institute trains PTSC facilitators on cardiovascular conditions, group facilitation and leadership skills, and the cultural beliefs undergirding self-management of chronic conditions. The facilitators are PTSC program graduates who live in the communities served by the program.

The PTSC program occurs over 13 weeks. Each facilitator-led Sister Circle includes 25 to 30 women. Each session lasts for 2 h and are held in accessible community sites (e.g., churches, public housing). Sister Circles are characterized by didactic training, guided discussions, and role-playing exercises that address CVD risk factors and strategies to overcome them. Facilitators follow a curriculum designed by the GPHIC that complies with The National Standards for Culturally and Linguistically Appropriate Services in Health and Health Care [17, 18]. The curriculum and its associated materials use text and illustrations to enhance knowledge uptake among women with varying literacy levels.

**Fig. 3:**
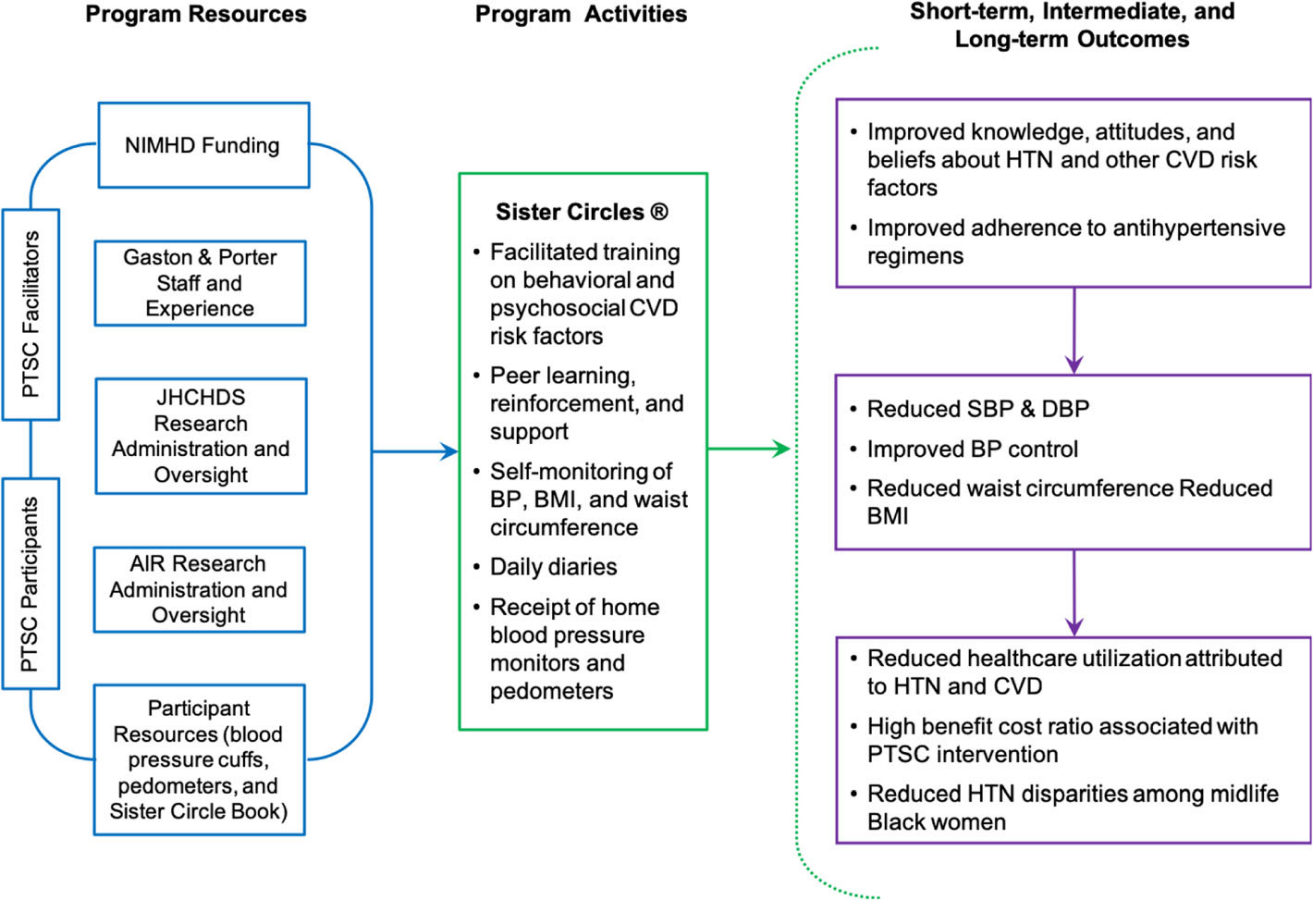
Logic Model for PTSC-RCT

The PTSC Program draws from three theoretical frameworks: the social cognitive theory [19], the transtheoretical model [20], and the Person, Extended Family, Neighborhood model [21]. Constructs from these models influence the PTSC Program’s underlying theory of change: by convening women in supportive Sister Circles led by a racially and socioeconomically concordant facilitator, participants will engage in peer learning and will initiate and maintain relationships with women in their community who are embarking on similar health journeys. This will lead to 1) improved knowledge, attitudes, and beliefs about hypertension and cardiovascular disease risk management, 2) improved self-efficacy related to adherence to pharmacological and lifestyle-based antihypertensive regimens, 3) individual, familial, and community capacity around hypertension prevention or management, and 4) improved blood pressure management among mid-life Black women, thereby reducing seemingly intransigent disparities observed within this population (explicated in Fig. 3). Its conceptual roots are consistent with empirical work documenting the importance of social support and social circles in promoting positive health changes among AA women [22–24].

### Study design

The PTSC study is a randomized controlled trial that aims to 1) estimate the effectiveness of PTSC compared with usual care on blood pressure control among hypertensive, low-income, mid-to-late life AA women; 2) estimate the effectiveness of PTSC on health knowledge, health self-efficacy, and health behaviors that contribute to risks associated with hypertension; and 3) test whether there is a cost offset of PTSC for society. The PTSC RCT was approved by the Institutional Review Boards (IRBs) of the Johns Hopkins Bloomberg School of Public Health (JHBSPH) and the American Institutes for Research (JHBSPH IRB No. 00007121).

### Hypotheses

We hypothesize that patients randomized to PTSC will have better-controlled blood pressure than patients who receive usual care at the end of the intervention (13 weeks) and up to one-year post-intervention (15 months). We expect patients randomized to the PTSC intervention to demonstrate a more significant increase in health knowledge, hypertension-related locus of control, physical activity, medication adherence, and stress management practices than patients receiving usual care. Finally, we anticipate that patients randomized to PTSC will have fewer hospitalizations and emergency rooms visits for cardiovascular-related events than patients receiving usual care, and that the costs of providing PTSC will be offset by reductions in healthcare costs associated with health improvements for patients randomized to PTSC, compared to patients receiving usual care.

### Study population and eligibility criteria

We plan to recruit up to 440 AA women receiving care at Unity Health Care (Unity), a Federally Qualified Health Center (FQHC) in Washington, D.C. and Baltimore Medical System Incorporated (BMSI), a FQHC with practices in Baltimore, Maryland. Eligible patient participants include English-speaking women who self-identify as AA, are 40 to 75 years old, have uncontrolled hypertension (previously defined as systolic blood pressure ≥ 140 mmHg or diastolic blood pressure ≥ 90) [2, 25], and receive their primary care at a Unity Health Care practice or a BMSI practice. Participants will be randomly assigned to receive the PTSC intervention or usual care at Unity or Baltimore Medical System Inc.

Thus far, we have recruited 341 participants into the study (PTSC intervention arm = 216; usual care arm = 125; see Fig. 1), all of whom receive care at Unity Health Care and live in metropolitan Washington D.C. In the next phase of the study, we will recruit an additional 100 AA women residing in Baltimore, Maryland and receiving care at a BMSI practice. Of note, hypertension diagnosis guidelines have changed since the study’s inception, and hypertension is now defined as systolic blood pressure ≥ 130 mmHg or diastolic blood pressure ≥ 80 [25]. However, we are maintaining the same blood pressure thresholds as we had at the start of the study in order to maintain equivalence in evaluating program impact between cohorts over time, given that the previous hypertension diagnosis criteria were central to the study’s eligibility criteria and recruitment process. Moreover, the patients are currently being managed to meet the prior guidelines. Our criteria reflect the current designation for Hypertension Stage 2 [25].

### Power calculation

In order to detect a statistically significant difference in blood pressure control between the intervention and control groups post-exposure to the PTSC intervention, we need 440 participants (*n* = 220 per group) in our study. Our power calculations were based on the assumption of 80% HTN prevalence among study participants in both treatment arms at baseline, a two-sided alpha of 0.05, and a 15% reduction in the proportion of participants with high blood pressure after receipt of the PTSC intervention. The calculated sample size was 308, but our experience developing, implementing, and evaluating interventions with this target population suggests that we should anticipate a relatively high attrition rate. Thus, we computed a potential attrition rate of roughly 40%. In order to have sufficient power to detect a 15% reduction in HTN in the intervention group, we will recruit at least 40% above the estimated sample size, for a total sample size of 440 participants between Unity and BMSI health systems.

### Study partners

This study is a collaboration between the Johns Hopkins School of Public Health Hopkins Center for Health Disparities Solutions (JHCHDS), the American Institutes for Research (AIR), the Gaston and Porter Health Improvement Center, Inc. (GPHIC), Unity Health Care (Unity), and Baltimore Medical System, Incorporated (BMSI). The PTSC Study was approved by the JHSPH and AIR Institutional Review Boards (IRBs). JHSPH HCHDS oversees recruitment and data collection for the PTSC study. AIR is responsible for data entry and management. GPHIC provides the PTSC program, materials, facilitators, and experts for those in the intervention group. Unity Health Care and BMSI assist with study recruitment through mass mailings to patients matching the eligibility criteria and posting flyers within centers. They also work directly with the PTSC-RCT partners to provide health care utilization data.

### Recruitment and randomization

Recruitment procedures commenced in 2017 and will cease in 2022. In the first wave of recruitment, Unity Health Care generated a list of the names and addresses of patients across their practices who fulfilled the study’s eligibility criteria. Candidate participants received a letter describing the study, incentives for participation, contact information for whom to follow up with, and the dates and locations for the recruitment sessions. We have also relied on word-of-mouth and targeted recruitment at Unity’s practices: Unity providers supplemented recruitment efforts by handing out flyers to eligible patients. We will follow the same recruitment processes for our BMSI participants.

Recruitment sessions, which occur in both the morning and afternoon of the same day to maximize attendance, are held in local churches located in predominantly AA neighborhoods. Women are randomly assigned to attend a session designed for the treatment arm or the usual care arm. JHCHDS staff, who conduct the randomization, are blinded to which sessions are for either arm, and both receive identical recruitment sessions. Each session features two interactive presentations. The first presentation, conducted by the GPHIC, describes cardiovascular health disparities at large and the ramifications of hypertension within AA communities, and highlights core components of the PTSC program. The second presentation, given by the JHCHDS, explains the study’s goals, timelines, and data collection procedures, and walks participants through the informed consent process. Attendees learn of their assigned treatment arm after the presentations have been made. At that point, written consent to participate in the study is obtained from those who wish to be a part of the trial.

### Baseline data collection

Upon consent, study participants complete a baseline survey composed of items measuring their health behaviors (including self-reported adherence, physical activity, and diet), psychosocial attributes (e.g., depressive symptomology, hypertension-related locus of control, and adaptive stress management techniques), perceived health status, and healthcare utilization.

Baseline measures of BP, waist circumference, height, and weight are also obtained. Blood pressure is assessed using a digital Omron 10 series device. Women are seated at rest for 5 min with their feet flat on the floor, and their arms raised at the heart level. Appropriate cuff sizes, based on each participant’s arm circumference, are attached to the device to detect their BP reading accurately.

### PTSC intervention

Members of the intervention group meet once a week over a 13-week period. During the first two-hour session, women learn about the program and discuss administrative details. In the remaining the Sister Circle sessions, facilitators and/or experts provide interactive educational presentations on topics related to high blood pressure and its management. These topics include stress management, inflammation, hypertension, and heart disease; depression and anxiety; self-esteem; physical activity and nutrition; strategies to partner with health care team members; and psychosocial issues unique to AA midlife women. Facilitators elicit participants’ challenges and successes from the previous week and use them as the basis for role-playing activities. This allows the women in the Sister Circle to work through specific scenarios based on the intersection of their personal circumstances and their attempts to adopt healthier behaviors.

Participants are also taught to accurately measure, record, and monitor their weight, body-mass-index (BMI), abdominal circumference, and blood pressure. They complete daily diaries and journals to log their behaviors and track their progress on achieving their health management goals. Participants receive $10 at each session to assist with transportation. They also receive the book, *Prime Time: The African American Woman’s Complete Guide to Midlife Health and Wellness,* [26] written by the co-developers of the PTSC Program, and relevant educational articles, self-assessments, home BP monitors, and pedometers. Upon completion of the PTSC program, participants officially graduate and obtain a certificate of completion from GPHIC, and a certificate of congratulations and gift from JHCHDS.

### Follow-up data collection

Follow-up surveys are completed at 13 weeks (end of the intervention), 9 months (6 months after the end of the intervention), and 15 months (a year after the end of the intervention). At the 15-month mark, those in the control group are eligible to enroll in the standard PTSC program. Study participants receive a $50 gift card each time they attend a data collection session. Participants who attend the recruitment session and all three follow-up sessions receive a total of $200. Administrative records from Unity are used to track healthcare utilization and costs over the study period. Thus far, two cohorts have enrolled in the study.

### Primary and secondary outcomes

The study’s primary outcomes are changes in blood pressure (baseline compared to 1-month post-intervention, 6 months post-intervention, and 1-year post-intervention); hospital utilization and admission, as well as emergency department utilization, attributable to cardiovascular events (obtained from Unity’s and BMSI’s medical records), and the intervention’s return on investment (which entails an interpolation of values from reported utilization from hospitalizations and ER, and lab and pharmacy visits, from Unity and BMSI). The secondary outcomes include changes in weight, BMI, and waist circumference; knowledge of causes and consequences of high blood pressure; hypertension-related locus of control; stress management techniques; self-reported physical activity, dietary behaviors, and blood pressure monitoring; and medication adherence. Data collection occurring at baseline, 13 weeks, 9 months, and 15 months post-intervention will capture blood pressure, weight, BMI, waist circumference, and the aforementioned secondary outcomes.

To assess participants’ knowledge about hypertension causes and consequences, we use the CAATCH Hypertension Knowledge Questionnaire, a 17-item questionnaire that requires to respondents to indicate if statements related to hypertension sequalae are true or false and is a modified version of a hypertension knowledge questionnaire developed by the National Heart, Lung, and Blood Institute [27, 28]. We measure hypertension-related locus of control, defined as a person’s beliefs concerning where control over their health lies, by modifying Form C of the Multidimensional Locus of Control Scale (specifically, we replaced “health” with. “hypertension” in each of the items) [29]. Stress management techniques are measured through a set of questions asking participants to report whether or not they are doing anything to lower their stress. If participants indicate “yes,” they are asked to mark which strategies they have employed to do so (all that apply). Responses include “listen to music or relaxation tapes,” “avoid it/forget about it,” “do yoga or tai chi,” and “talk with professional counselor or therapist.” Self-reported physical activity is evaluated through questions asking whether or not participants engaged in at least 30 min of exercise during the week. If they answered “yes,” they were asked to indicate what type(s) of exercises they did (e.g., walking, dancing, swimming). We elicit dietary behaviors through questions that asked how participants eat in a typical day; participants indicated whether or not they did such things as adding salt to their food, consuming fast food, or eating when they are upset. Blood pressure self-monitoring is encapsulated through questions that asked whether or not participants have taken their own blood in the last 12 months and, if so, if they have done so daily, weekly, or monthly. Finally, medication adherence is measured through the 9-item Hill-Bone Medication Adherence scale [30]. Scores range from 9 to 36, with higher scores reflecting poorer adherence to antihypertensive pharmacological therapies [31].

### Analysis plan

Study data will be exported into Stata 15 for statistical analysis. We will set statistical significance at *p* ≤ 0.05. Baseline descriptive characteristics and outcomes will be analyzed using mean and standard deviation (SD) for continuous data, and counts and percentages for categorical data**.** We will conduct unequal variances t-tests in order to detect statistically significant differences between the treatment arms. To assess the impact of the PTSC program on participants’ blood pressure, weight, BMI, waist circumference, and all of the secondary outcomes, we will employ a difference-in-differences methodology to observe changes in participants’ outcomes at 3 months, 6 months, and 12 months post-intervention. We will also use multivariable regression analyses to discern whether or not participants’ outcomes improved as a result of receiving the PTSC intervention. Our models will control for patients’ sociodemographic characteristics (including age, marital status, and income), health insurance status, substance use (namely, smoking and drinking), and the presence of chronic conditions.

## Discussion

Preliminary analyses of the baseline characteristics of the study’s participants reveal.

that, thus far, a substantial portion of participants assigned to the intervention group did not receive the intervention as intended. Of the 216 women randomly assigned to the PTSC intervention, 103 received the intervention as designed (that is, they attended at least 8 of the 13 Sister Circles), while the remaining 113 did not. This indicates a need for culturally- and economically-sensitive mitigation strategies that will encourage higher receipt of the intervention. In an effort to uncover some of the factors related to low exposure to the PTSC program, we created a short survey to elicit information about why they were not participating. Two of the top 3 reasons cited for non-involvement in the study were related to the accessibility of the Sister Circles (transportation and scheduling), and the other reason was a matter of personal health (not feeling well enough to attend). These realities attest to the difficulties associated with designing and implementing a randomized trial in the real-world context of engaging with a socioeconomically vulnerable study population, whose members may be grappling with multiple comorbid conditions and various structural determinants of health. Additional analyses indicate that most of the attrition observed thus far occurred between the recruitment meeting and participants’ first Sister Circle session, despite the fact that participants meet their facilitator at the recruitment meeting, and facilitators contacted study participants in between the recruitment session and the first Sister Circle session. Bearing in mind that the reduced number of study participants exposed to the intervention threatens our ability to detect statistically significant differences between the intervention and usual care groups, and to tackle the issue of attrition prior to the first Sister Circle, we have shortened the time between the recruitment meeting and the first Sister Circle session to less than two weeks. This information informs our approach for the third cohort of participants. We will make the Sister Circle schedules more flexible, provide transportation assistance, and create protocols for facilitators to follow up with participants prior to the start of their Sister Circle. In addition, we will allow study participants assigned to the intervention to choose their Sister Circle from a menu of Sister Circles that vary by time, i.e., from a daytime, evening, and weekend Sister Circle.

Despite recruitment challenges, the PTSC-RCT joins a growing body of rigorously-evaluated community-based interventions geared toward reducing disparities in blood pressure control among AA women. They are typified by a blend of peer support and didactic training and education, the combination of which holds promise in addressing psychosocial and behavioral factors contributing to poorly controlled hypertension [8, 32–35]. These studies suggest that the cultivation of gender-specific, culturally concordant peer networks may shift underlying attitudes and beliefs about hypertension and its management, reinforce proper education about hypertension causes and treatment, and provide support and encouragement for adherence to antihypertensive regimens.

The overarching goal of the PTSC RCT is to determine if AA women with hypertension participating in the PTSC intervention exhibit improved blood pressure control; knowledge, attitudes, and beliefs about hypertension and its management; adherence to antihypertensive regimens; and psychosocial well-being; as well as reduced CVD risk factors and health care utilization stemming from CVD. We will also discern the program’s return on investment, an understudied area in the context of community-based interventions addressing disparities in cardiovascular disease. Indeed, the PTSC RCT presents an important opportunity to demonstrate the effectiveness of a longstanding community-based program emerging from the work and expertise of two AA women. There is indication to suggest that the conceptual underpinnings of the intervention, and its core elements, are associated with reductions in cardiovascular disease risk factors among AA women. Consequently, this study will illuminate the constellation of real-world barriers associated with designing and implementing randomized controlled trials among members of a highly vulnerable population, which is essential for identifying and advancing best-practices for reducing racial/ethnic disparities in cardiovascular health.

## Data Availability

The datasets used and/or analyzed during the current study are available from the corresponding author on reasonable request.

## Abbreviations

AA: African American
AIR: American Institutes for Research
BMI: Body mass index
BMSI: Baltimore Medical System, Incorporated
CVD: Cardiovascular disease
FQHC: Federally Qualified Health Center
GED: General Educational Development
GPHIC: Gaston and Porter Health Improvement Center, Inc.
ITT: Intent-to-treat
JHCHDS: Johns Hopkins Center for Health Disparities Solutions
PTSC: Prime Time Sister Circle
RCT: Randomized controlled trial

## References

[1] Fryar CD, Ostchega Y, Hales CM, Zhang G, Kruszon-Moran D (2017). Hypertension prevalence and control among adults: United States, 2015-2016. NCHS Data Brief.

[2] Chobanian AV, Bakris GL, Black HR, et al. The seventh report of the joint national committee on prevention, detection, evaluation, and treatment of high blood pressure: The JNC 7 report. *JAMA*. 2003;289(19):2560–2572. doi: 10.1001/jama.289.19.2560 [doi]. 10.1001/jama.289.19.2560 12748199

[3] Danaei G, Ding EL, Mozaffarian D, et al. The preventable causes of death in the united states: Comparative risk assessment of dietary, lifestyle, and metabolic risk factors. *PLoS Med*. 2009;6(4):e1000058. doi: 10.1371/journal.pmed.1000058 [doi]. 10.1371/journal.pmed.1000058 PMC2667673 19399161

[4] Egan BM, Zhao Y, Axon RN (2010). US trends in prevalence, awareness, treatment, and control of hypertension, 1988-2008. JAMA..

[5] Chen V, Ning H, Allen N (2019). Lifetime risks for hypertension by contemporary guidelines in African American and White men and women. JAMA Cardiol.

[6] Go AS, Mozaffarian D, Roger VL (2014). Executive summary: heart disease and stroke statistics--2014 update: a report from the American Heart Association. Circulation..

[7] Hill MN, Bone LR, Kim MT, Miller DJ, Dennison CR, Levine DM (1999). Barriers to hypertension care and control in young urban black men. Am J Hypertens.

[8] Rodriguez F, Christopher L, Johnson CE, Wang Y, Foody JM (2012). Love your heart: a pilot community-based intervention to improve the cardiovascular health of African American women. Ethn Dis..

[9] Schoenthaler AM, Lancaster KJ, Chaplin W, Butler M, Forsyth J, Ogedegbe G (2018). Cluster randomized clinical trial of FAITH (FAITH-based approaches in the treatment of hypertension) in blacks. Circ Cardiovasc Qual Outcomes..

[10] White BM, Rochell JK, Warren JR (2020). Promoting cardiovascular health for African American women: an integrative review of interventions. J Women's Health (Larchmt).

[11] Schneider RH, Staggers F, Alxander CN (1995). A randomised controlled trial of stress reduction for hypertension in older African Americans. Hypertension..

[12] Feathers JT, Kieffer EC, Palmisano G (2007). The development, implementation, and process evaluation of the REACH Detroit Partnership's diabetes lifestyle intervention. Diabetes Educ.

[13] Flack JM, Sica DA, Bakris G (2010). Management of high blood pressure in blacks: an update of the international society on hypertension in blacks consensus statement. Hypertension..

[14] Shaya FT, Gu A, Saunders E (2006). Addressing cardiovascular disparities through community interventions. Ethn Dis..

[15] Gaston MH, Porter GK, Thomas VG (2007). Prime time sister circles: evaluating a gender-specific, culturally relevant health intervention to decrease major risk factors in mid-life African-American women. J Natl Med Assoc.

[16] Thomas VG, Gaston MH, Porter GK, Anderson A (2016). Prime time sister circles(®)II: evaluating a culturally relevant intervention to decrease psychological and physical risk factors for chronic disease in mid-life African American women. J Natl Med Assoc.

[17] Narayan MC (2001). The national standards for culturally and linguistically appropriate services in health care. Care Manag J.

[18] IQ Solutions, Inc. National Standards for Culturally and Linguistically Appropriate Services in Health Care. U.S. Department of Health and Human Services Office of Minority Health (March 2001). https://minorityhealth.hhs.gov/assets/pdf/checked/finalreport.pdf. .

[19] Bandura A (1986). Social foundations of thought and action. Englewood Cliffs, NJ.

[20] Prochaska JO, Velicer WF (1997). The transtheoretical model of health behavior change. Am J Health Promot.

[21] Airhihenbuwa CO (1992). Health promotion and disease prevention strategies for African Americans: a conceptual model. Health issues in the Black community.

[22] Webb MS, Gonzalez LO (2006). The burden of hypertension: mental representations of African American women. Issues Ment Health Nurs.

[23] Drayton-Brooks S, White N (2004). Health promoting behaviors among African American women with faith-based support. ABNF J.

[24] Geller JL, Salzer MS (1997). Support groups: current perspectives on theory and practice. Psychiatr Serv.

[25] Whelton PK, Carey RM, Aronow WS (2018). 2017 ACC/AHA/AAPA/ABC/ACPM/AGS/APhA/ASH/ASPC/NMA/PCNA guideline for the prevention, detection, evaluation, and management of high blood pressure in adults: a report of the american college of cardiology/american heart association task force on clinical practice guidelines. J Am Coll Cardiol.

[26] Gaston MH, Porter GK. Prime time: the African American woman's complete guide to midlife health and wellness. One World/Ballantine; 2003.

[27] Ogedegbe G, Tobin JN, Fernandez S (2009). Counseling African Americans to control hypertension (CAATCH) trial: a multi-level intervention to improve blood pressure control in hypertensive blacks. Circ Cardiovasc Qual Outcomes.

[28] Carter-Edwards L, Jackson SA, Runaldue MJ, Svetkey LP (2002). Diet- and blood pressure-related knowledge, attitudes, and hypertension prevalence among African Americans: the KDBP study. Knowledge of diet and blood pressure. Ethn Dis.

[29] Wallston KA, Stein MJ, Smith CA (1994). Form C of the MHLC scales: a condition-specific measure of locus of control. J Pers Assess.

[30] Kim MT, Hill MN, Bone LR, Levine DM (2000). Development and testing of the Hill-Bone compliance to high blood pressure therapy scale. Prog Cardiovasc Nurs.

[31] Song Y, Han HR, Song HJ, Nam S, Nguyen T, Kim MT (2011). Psychometric evaluation of hill-bone medication adherence subscale. Asian Nurs Res (Korean Soc Nurs Sci).

[32] Ard JD, Carson TL, Shikany JM (2017). Weight loss and improved metabolic outcomes amongst rural African American women in the deep south: six-month outcomes from a community-based randomized trial. J Intern Med.

[33] Blanks SH, Treadwell H, Bazzell A (2016). Community engaged lifestyle modification research: engaging diabetic and Prediabetic African American women in community-based interventions. J Obes.

[34] Kang AW, Dulin A, Nadimpalli S, Risica PM. Stress, adherence, and blood pressure control: A baseline examination of black women with hypertension participating in the SisterTalk II intervention. *Prev Med Rep*. 2018;12:25–32. doi: 10.1016/j.pmedr.2018.08.002 [doi]. 10.1016/j.pmedr.2018.08.002 PMC6098218 30128268

[35] Villablanca AC, Warford C, Wheeler K (2016). Inflammation and Cardiometabolic risk in African American women is reduced by a pilot community-based educational intervention. J Women's Health (Larchmt).

